# 

**Published:** 1884-08

**Authors:** 


### CONTENTS.

### COMMUNICATIONS.

Dental Education........ 369
The Causes of Failure in Dental Operations........ 377
My Failures........ 382
The Promotion of Osseous Development........ 397
Antiseptics and Disinfectants........ 402

SELECTIONS.

Case of Spasm of Muscles of Face and Cataract Due to Dental Irritation........ 389
The Hippocratic Oath........ 392
Small Doses Frequently Repeated........ 393
The Hypodermic Injection........ 395
Feeding Infants........ 395
A Bar to Malpractice Suits........ 395
Removal of Warts........ 396
Tongaline........ 396
The Writing of Galen........ 396

CORRESPONDENCE.

Letter from London........ 407
Letter from Geneva........ 409

SALMAGUNDI........ 411

EDITORIAL........ 418
Minnesota State Dental Society........ 418
Extension of Term........ 418
Indiana Dental College........ 419
To Saratoga........ 419
Lost.—Registers........ 420
Married........ 420

